# Type-entry-malperfusion classification in a 13-year cohort of surgically treated type A acute aortic syndromes: Impact on planning and outcomes

**DOI:** 10.1016/j.xjon.2026.101614

**Published:** 2026-02-06

**Authors:** Jacques Tomasi, Pierre Escrig, Jean Philippe Verhoye

**Affiliations:** Department of Cardiothoracic Surgery, Pontchaillou University Hospital, Rennes, France

**Keywords:** acute aortic syndrome, type A dissection, TEM classification

## Abstract

**Objectives:**

The Stanford classification helps rapidly triage patients with type A acute aortic syndrome, but it overlooks key elements like proximal entry tear and malperfusion. The newer type, entry, malperfusion classification addresses these gaps to improve management.

**Methods:**

We conducted a retrospective monocentric study of all patients operated for type A acute aortic syndrome between 2010 and 2023.

**Results:**

Among 334 included patients, hospital mortality was 16.5%. Entry tear was located in the ascending aorta in 69%, the arch in 19%, and the descending aorta in 3.6%. Malperfusion occurred in 75% of patients; 35% had at least 1 clinical malperfusion. Arch or distal entry tears were significantly associated with more extensive arch resections. Coronary malperfusion led to more associated procedures (coronary artery bypass grafting, reimplantation), more frequent root replacements (M1−), and greater extracorporeal life support use (M1+). Clinical malperfusions were associated with greater mortality and complication rates. Patients who were M3+ displayed the most severe multiorgan complications.

**Conclusions:**

The type, entry, malperfusion classification offers valuable preoperative insights, helping to anticipate surgical strategy and identify high-risk patients. However, its ability to differentiate malperfusion severity remains limited.

## Graphical Abstract

**Figure:**
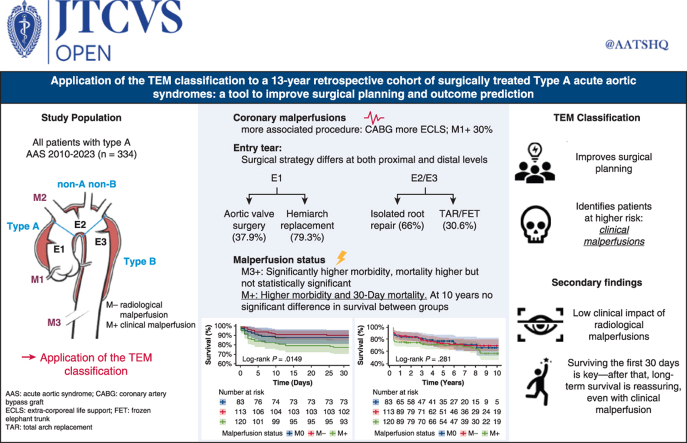

**Figure:**
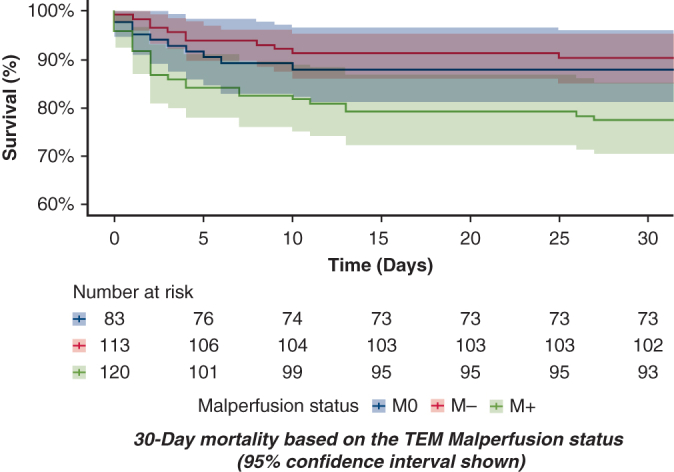
30-day mortality based on the TEM malperfusion status (95% CI shown).

**Central Message**

The TEM classification applied to acute aortic syndromes may improve surgical decision-making and better stratify patients according to malperfusion status to identify postoperative high-risk patients.

**Perspective**

The TEM classification addresses anatomical items and helps surgeons to anticipate the appropriate surgical approach, with more complex arch resections with entry tear in the arch of further. It identifies high-risk patients, especially those with clinical malperfusions, but lacks discriminative power between symptoms of the same topography with different clinical implications.

Acute aortic syndromes (AAS) are life-threatening emergencies, with mortality rates reaching 1 to 2% per hour for the first 48 hours.^1^ The Stanford classification,^2^ in use for more than 50 years, is simple and efficiently guides urgent surgical treatment by distinguishing type A (ascending aorta involvement) from type B (descending aorta only). The Stanford system does not account for key prognostic elements, such as malperfusion syndromes, which are present in 20 to 30% of patients^3^ and contribute substantially to AAS-related morbidity and mortality.

Recently, major progress has been made in aortic surgery with the development of more advanced imaging modalities, new hybrid prostheses, endovascular techniques, and cerebroprotection, enabling tailored treatment of AAS and requiring a more precise description of the pathology. The TEM classification was recently proposed by Sievers and colleagues.^4^ Built upon the Stanford classification, it introduces the type nonA-nonB when the aortic arch is compromised but not the ascending aorta and adds 2 critical parameters:

- The entry tear “E,” that has to be removed during surgery as recommended in the latest European Association for Cardio-Thoracic Surgery recommendations.^5^

- The malperfusion status “M,” stratified by territory M1/coronary, M2/supra-aortic, and M3/spinal; visceral, renal, and iliac; and by clinical involvement such as M− for radiologic malperfusions and M+ for symptomatic or biological malperfusion.

Endorsed by the latest European guidelines, the TEM system aims to improve initial surgical planning and outcome prediction, yet its real-world applicability remains insufficiently explored. In this study, we aim to analyze our results on the management of type A AAS in our tertiary center and to apply the new TEM classification to evaluate its utility in guiding surgical strategy and identifying high-risk patients.

## Methods

We retrospectively reviewed all patients who underwent surgery for acute type A AAS between January 1, 2010, and December 31, 2023, at our institution. Patients who died before surgery or were managed conservatively were excluded. Patients with symptom/dissection onset less than 14 days were considered as acute.

This study was conducted in accordance with the Declaration of Helsinki and complied with the General Data Protection Regulation. According to French law, retrospective observational studies based exclusively on existing data and involving no intervention do not require approval from an institutional review board. Accordingly, the requirement for individual ethics committee approval was waived. The institutional database used for this study was declared to and approved by the French National Commission for Data Protection (CNIL; authorization number 1685088, July 25, 2013). Written informed consent for the use of medical data for research purposes was obtained from all patients. Computed tomography scans were reviewed using multiplanar reconstructions perpendicular to the aortic axis. Survival was verified through the French national registry (Institut National de la Statistique et des Etudes Econonomiques). TEM classification was performed according to Sievers and colleagues.

All surgeries were performed via median sternotomy using antegrade cold crystalloid cardioplegia. Decisions regarding cannulation site, circulatory arrest, cerebral protection, and aortic repair were at the discretion of the operating surgeon. Isolated root repair consisted of conservative techniques of the root involving commissure resuspensions and the use of biological glue to reapproximate the dissected aortic layers.

Continuous variables are expressed as mean ± standard deviation or median [interquartile range] and categorical variables as counts and percentages. For statistical comparisons, we used χ^2^, Fisher exact, t test, Wilcoxon rank-sum, or binomial tests, as appropriate. Analysis of variance or Kruskal-Wallis tests were used for multiple group comparisons. Survival was analyzed using Kaplan-Meier estimates and log-rank tests. Analyses were performed on available cases without imputation. All analyses were performed using R, version 4.4.0 (R Foundation for Statistical Computing).

## Results

**Figure E1:**
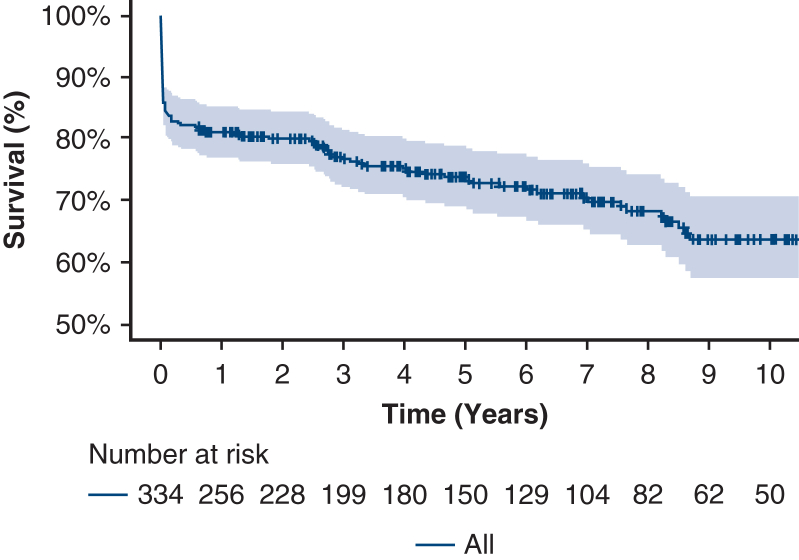
Kaplan-Meier survival curve. 95% confidence limits are shown.

A total of 334 patients were included. Their baseline characteristics are summarized in Table 1. Preoperative status, perioperative data, postoperative, and follow-up details are shown in Table E1, Table E2, Table E3, Table E4. Thirty-day mortality was 15.8%. One-, 5-, and 10-year survival was 81%, 73.7%, and 59.7%, respectively (Figure E1). No significant difference in aortic reintervention was observed between hemiarch and total arch replacement (either technique) (7.1% vs 6.2%, *P* value = 1.0) (Table E5). The effective mortality compared with the predicted mortality by the German Registry of Acute Aortic Dissection type A score (Table E6) was lower in each subgroup but with no significant difference.

**Table 1:** Patient characteristics

Variable	N = 334
Sex (male)	223 (67)
Age, y	63.0 ± 12.4
Body mass index, kg/m^2^	26.2 ± 4.8
Hypertension	206 (62)
Smoking status	
None	230 (69)
Active	67 (20)
Stopped	36 (11)
Dyslipidemia	69 (21)
Obesity	68 (21)
Diabetes	14 (4.3)
Chronic kidney disease	12 (3.6)
Heredity	20 (6.1)
Respiratory pathology	29 (8.8)
Cancer	14 (4.2)
Peripheral artery disease	11 (3.3)
Redux	8 (2.4)
Type B dissection	8 (2.4)
Ischemic cardiomyopathy	14 (4.2)
Arrhythmia	107 (33)
Inflammatory disease	9 (2.7)
Connective tissue disorder	20 (6.0)
Marfan	16 (4.8)
Turner	3 (0.9)
Loeys-Dietz	1 (0.3)

Values are n (%) or mean ± standard deviation.

Although all cases were considered before the study as type A, 5 patients were classified as nonA-nonB, and no patients with type B were identified (Table 2). The proximal entry tear was in the ascending aorta in 69% of patients, in the aortic arch in 19%, and in the descendant thoracic aorta in 3.6%. For 26 patients (7.8%), no entry tear was identified. Almost 75% had a malperfusion of any kind, most commonly in the M3 territory, followed by M2, and coronary malperfusions were observed in 15% of the patients. Considering all types of malperfusion, 27% of patients had 1, 38% had 2, and 8.9% had 3 malperfusions. Clinical malperfusion was present in 1 territory in 28.4%, in 2 territories in 7.5%, and none had all 3 territories involved.

**Table 2:** TEM description

Characteristic	N = 334
T	
A	329 (98)
B	
NonA-nonB	5 (1.5)
E	
Missing data	5 (1.5)
0	26 (7.9)
1	228 (69)
2	63 (19)
3	12 (3.6)
M	
Missing data	18 (5.4)
M1	
0	265 (84)
1−	25 (7.9)
1+	26 (8.2)
M2	
0	137 (43)
2−	112 (35)
2+	67 (21)
M3	
0	134 (42)
3−	131 (41)
3+	51 (16)
Number of malperfusion territories involved	
0	83 (26)
1	86 (27)
2	119 (38)
3	28 (8.9)
Number of radiologic malperfusion	
0	132 (42)
1	111 (35)
2	67 (21)
3	6 (1.9)
Number of clinical malperfusion	
0	196 (62)
1	95 (30)
2	25 (7.9)

Values are n (%).

Entry tear location (Table 3) influenced proximal repair strategy: more aortic valve replacements were performed in E1, whereas E2 and E3 were more likely to receive isolated root repairs (*P* value .003). Distal aortic strategy also varied significantly: hemiarch was preferred in E1, whereas total arch replacement and frozen elephant trunk were more frequent in E2/E3 (*P* < .001). Circulatory arrest and duration were greater in E2/E3 (*P* .010 and *P* < .001). Although trends suggested different cerebral protection strategies across groups, statistical significance was not reached (*P* .067). No mortality or stroke differences were observed between entry tear groups.

**Table 3:** Surgical characteristics and results based on the level of the entry tear

Variable	E1 (n = 228)	E2 (n = 63)	E3 (n = 12)	*P* value∗,†
Cardiopulmonary bypass time, min	177.4 ± 55.3	209 ± 71.5	175.9 ± 62.1	**.001**
Crossclamp time, min	122 ± 44	133.1 ± 49.1	132.3 ± 50.8	.192
Arterial canulation				.6
Femoral	119 (52.4)	27 (43.5)	8 (66.7)	
Axillary	73 (32.2)	25 (40.3)	3 (25.0)	
Central	31 (13.7)	9 (14.5)	1 (8.3)	
Other (RCC, innominate, etc)	4 (1.7)	1 (1.6)	0 (0)	
Circulatory arrest	198 (86.8)	62 (98.4)	12 (100.0)	**.010**
Arrest temperature	24 ± 4.4	23.9 ± 3.7	23.5 ± 3.4	.918
Circulatory arrest time	33 ± 15	49.9 ± 26.3	57.5 ± 26.1	**<.001**
Type of cerebral perfusion				.067
Deep hypothermia only	70 (35.5)	14 (22.6)	1 (8.3)	
Retrograde	1 (0.5)	1 (1.6)	0 (0)	
Antegrade unilateral	41 (20.8)	14 (22.6)	4 (33.3)	
Antegrade bilateral	84 (42.6)	32 (51.6)	6 (50.0)	
Retrograde and antegrade	1 (0.5)	1 (1.6)	1 (8.3)	
Proximal aortic repair				**.003**
No repair	4 (1.8)	7 (11.3)	2 (16.7)	
Aortic valve replacement	24 (10.6)	3 (4.8)	1 (8.3)	
Isolated root repair	136 (59.9)	41 (66.1)	9 (75.0)	
Bentall	46 (20.3)	11 (17.7)	0 (0)	
Tirone David	16 (7.0)	0	0 (0)	
Florida sleeve	1 (0.4)	0	0 (0)	
Distal aortic repair				**<.001**
Hemiarch	180 (79.3)	42 (67.7)	9 (75.0)	
TAR	6 (2.6)	8 (12.9)	2 (16.7)	
FET	5 (2.2)	12 (19.4)	1 (8.3)	
Limited ascending aortic replacement	34 (15.0)	0 (0)	0 (0)	
Wrapping	2 (0.9)	0 (0)	0 (0)	
Postoperative death	37 (16.2)	10 (15.9)	1 (8.3)	>.9
Stroke	30 (13.5)	11 (18.6)	2 (16.6)	.3

Values are n (%) or mean ± standard deviation. Bolded *P* values indicate statistical significance (*P* < .05). *RCC*, Right common carotid; *TAR*, total arch replacement; *FET*, frozen elephant trunk; *ANOVA*, analysis of variance.

∗ Fisher exact test for categorical variables.

† ANOVA test for continuous variables.

Coronary malperfusion status (M1) significantly affected surgical strategy (Table 4). Bentall procedures were more frequent in patients who were M1 (52%, *P* = .048). Associated procedures, particularly coronary interventions, were more common in M1− and M1+ (*P* = .001 and *P* = .002). Extracorporeal life support was used more often in patients who were M1+ (19.2%, *P* = .030). Although not statistically significant, mortality was greater in the M1+ group (23%) (Table E7).

**Table 4:** Proximal surgery based on the coronary malperfusion status

Characteristics	M1-0 N = 265	M1− N = 25	M1+ N = 26	*P* value∗
Proximal aortic repair				**.048**
No proximal repair performed	13 (5.0)	0 (0)	0 (0)	
Aortic valve replacement	26 (9.9)	2 (8.0)	2 (7.7)	
Isolated root repair	166 (63.4)	10 (40.0)	16 (61.5)	
Bentall	44 (16.8)	13 (52.0)	6 (23.1)	
Tirone David	11 (4.2)	0 (0)	2 (7.7)	
Florida sleeve	2 (0.8)	0 (0)	0 (0)	
Associated procedure	24 (9.1)	6 (24.0)	8 (30.8)	**.001**
Type of associated procedure				**.002**
None	238 (90.8)	19 (76.0)	18 (69.2)	
CABG or coronary reimplantation	18 (6.9)	5 (20.0)	8 (30.8)	
Other (MVR, tricuspid repair, etc)	6 (2.3)	1 (4.0)	0 (0)	
ECLS	14 (5.3)	2 (8.0)	5 (19.2)	**.030**

Values are n (%). Bolded *P* values indicate statistical significance (*P* < .05). *CABG*, Coronary artery bypass grafting; *MVR*, mitral valve replacement; *ECLS*, extracorporeal life support.

∗ Fisher exact test.

Stroke occurred more frequently in M2− and M2+ (16% and 18% vs 10%), although only intensive care unit (ICU) stay differed significantly (*P* .031). In M3 malperfusion, clinical forms (M3+) were associated with significantly longer hospital and ICU stays (*P* = .005 and *P* = .004), increased renal failure (67%, *P* < .001), infections (48%, *P* = .002), and visceral complications (17%, *P* = .001). Mortality was greater in M3+ (25%) but did not reach statistical significance (*P* = .13).

Patients with ≥2 malperfusions had longer hospital and ICU stays (*P* = .004 and < .001, Table 5), with more visceral complications (*P* = .024) and infection (*P* = .006). Mortality was also greater but not statistically significant. When stratified by the number of radiologic malperfusions, only the time of hospitalization and in the ICU showed significant differences (*P* = .02 and .046).

**Table 5:** Outcomes based, respectively, on the total number of malperfusions, the number of radiological malperfusions, and the number of clinical malperfusion syndromes

Number of malperfusions	0 N = 83∗	1 N = 86	≥2 N = 147	*P* value∗
Time of hospitalization, d	17.8 ± 22.0	16.9 ± 13.4	20.9 ± 14.6	**.004**
Time in the ICU, d	8.6 ± 21.3	6.5 ± 5.5	11.0 ± 11.2	**<.001**
Stroke	11 (14)	7 (8.4)	24 (17)	.2
Renal failure	33 (41)	29 (35)	73 (51)	.054
Visceral complications	1 (1.3)	2 (2.4)	13 (9.0)	**.024**
Pulmonary complications	14 (18)	14 (17)	39 (27)	.12
Infection	17 (22)	20 (24)	57 (40)	**.006**
Death	11 (13)	13 (15)	26 (18)	.7

Number of radiologic malperfusions	0 N = 132	1 N = 111	≥2 N = 73	*P* value∗
Time of hospitalization, d	17.3 ± 19.2	20.6 ± 15.2	19.5 ± 13.1	**.020**
Time in the ICU, d	8.4 ± 17.5	9.8 ± 10.7	9.5 ± 9.4	**.046**
Stroke	14 (11)	19 (18)	9 (13)	.4
Renal failure	54 (43)	49 (45)	32 (45)	>.9
Visceral complications	4 (3.2)	6 (5.6)	6 (8.3)	.3
Pulmonary complications	25 (20)	29 (27)	13 (18)	.3
Infection	33 (26)	36 (33)	25 (35)	.3
Death	24 (18)	14 (13)	12 (16)	.5

Number of clinical malperfusion	0 N = 196	≥1 N = 120	*P* value†
Time of hospitalization, d	18.5 ± 17.3	19.8 ± 15.4	.3
Time in the ICU, d	8.4 ± 15.1	10.3 ± 11.0	.11
Stroke	23 (12)	19 (16)	.3
Renal failure	75 (39)	60 (52)	**.044**
Visceral complications	6 (3.2)	10 (8.6)	.061
Pulmonary complications	34 (18)	33 (28)	**.046**
Infection	48 (25)	46 (40)	**.011**
Death	23 (12)	27 (23)	**.017**

Values are n (%) or mean ± standard deviation. Bolded *P* values indicate statistical significance (*P* < .05). *ICU*, Intensive care unit.

∗ Kruskal-Wallis rank sum test; Fisher exact test.

† Wilcoxon rank sum test; Fisher exact test.

**Figure 1:**
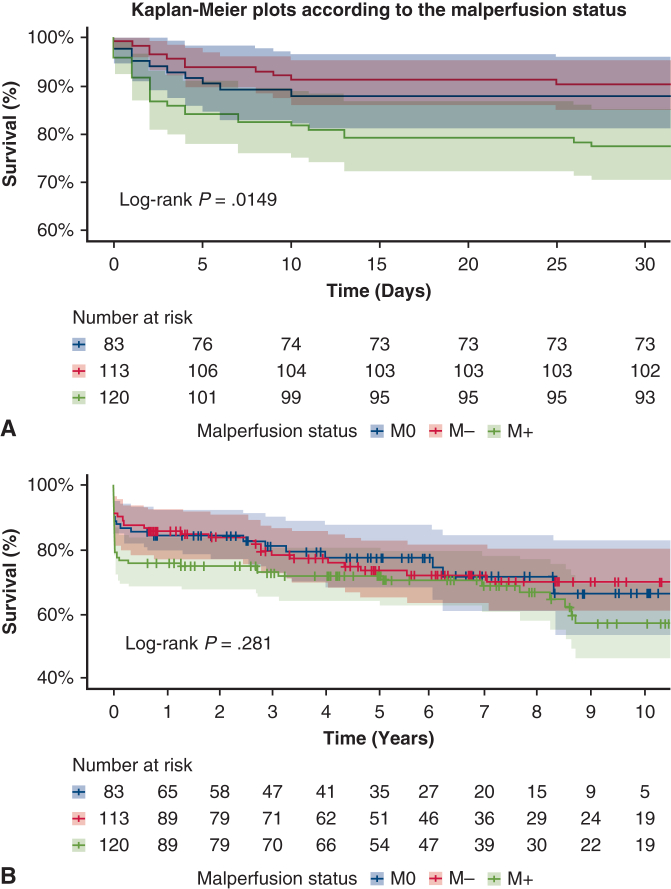
Kaplan-Meier survival curves stratified by malperfusion status M0/M− and M+. A, Early survival at 30 days. B, Long-term survival up to 10 years. 95% confidence limits are shown.

Patients with at least 1 clinical malperfusion had significantly greater mortality than those without (23% vs 12%, *P* = .017) and more infections (*P* = .011), pulmonary complications (*P* = .046), and renal failure (*P* = .044). Visceral complications showed a trend toward significance (*P* = .061). Patients who were M+ had significantly lower 30-day survival (*P* = .0149) compared with patients who were M0 and M−. At 10 years, survival remains lower but not statistically significant (Figure 1).

## Discussion

The Stanford classification has been the standard for more than 50 years. Its simplicity allows a clear communication between general practitioners/emergency department personnel, with surgeons assuring a quicker triage of the patients, and still should be the only classification used at this level. However, it lacks crucial information on disease extent, entry tear location, and malperfusion, key elements for surgical planning and prognosis.

Alternative classifications each have strengths and limitations. The DeBakey score^6^ offers anatomical precision but lacks emergency practicality. DISSECT^7^ is comprehensive yet too complex for urgent care. The Society for Vascular Surgery/Society of Thoracic Surgeons system^8^ focuses on entry site and extent but may reclassify classic type A as type B. The Penn classification^9^ improves risk stratification for malperfusion, with some predictive value for 30-day mortality,^10^ but omits anatomical information.

The TEM classification fills these gaps, combining dissection type, entry tear site, and malperfusion status. Endorsed in recent guidelines, its clinical validation is limited. Our study provides real-world evidence from a 13-year cohort of 334 surgically treated patients, demonstrating its utility for surgical planning and outcome prediction. With a 16.5% hospital mortality in accordance with the literature and lower-than-predicted mortality based on the German Registry of Acute Aortic Dissection type A score^11^ in each subgroup, those results tend to validate our strategies.

### The “T”: Relevance of NonA-NonB

NonA-nonB dissections, representing ∼10% of AAS^12^ cases, remain debated: should they be treated as type A, type B, or a distinct entity? The literature, based on small cohorts, diverges on optimal strategies: frozen elephant trunk, thoracic endovascular aneurysm repair, or hybrid procedures.^13^ Our series only reported 5 patients with nonA-nonB, probably because they were managed medically, such as a type B. We could not draw any conclusion from this subgroup because of its very small size, but using the TEM routinely would allow the identification of them and potentially aid in management standardization with bigger cohorts.

### Entry Tear Localization and Surgical Strategy

The ideal extent of aortic resection remains debated. One approach favors simple, faster surgeries with possible staged interventions.^14^ Others advocate immediate extensive arch replacement to minimize reintervention risk.

Arch replacement offers a circular distal anastomosis, less prone to dilation than hemiarch, reducing distal anastomosis new entry risks.^15^ Its distal location also facilitates endovascular treatments. In our study, entry tear location significantly influenced surgical strategy: more arch replacements and longer circulatory arrests were observed in patients who were E2/E3, who also benefitted more frequently (but not significantly) of a cerebral protection with cerebroperfusion. However, stroke and mortality rates were not significantly different than those of patients who were E1, supporting anatomically tailored approaches. Despite entry tears in the arch, approximatively 70% of our patients underwent hemiarch resection when sufficient to remove the entry tear. This conservative strategy was effective, with low reintervention rates and similar outcomes observed compared with more extensive repairs, in line with existing reports.^16^

### Malperfusions as a Key Prognostic Factor

Malperfusion critically impacts outcomes: patients may succumb to ischemic injury despite successful aortic repair.^17^ The classic paradigm promotes urgent aortic surgery to restore true lumen flow, which is the strategy performed in our center. Some teams, in cases of hemodynamically stable patients presenting with an organ malperfusion, treat the malperfusion first using endovascular techniques (fenestration, stenting) and then perform the aortic surgery.^18^

The Michigan team^19^ reported a 30-day mortality of only 3.7% in patients initially treated for malperfusion before definitive aortic surgery, which is significantly lower than the expected mortality for any upfront patient with an AAS. The risk of dying from aortic rupture in the meantime was 6.6-fold lower than dying of organ failure.

Coronary malperfusion (M1+) was associated with greater extracorporeal life support use and a trend toward increased mortality, reflecting the severity of myocardial ischemia and potential suboptimal myocardial protection. Proximal repairs were more complex in these patients, with more frequent coronary artery bypass graft and Bentall procedures.

Patients with at least 1 clinical malperfusion (M+), regardless of territory, had significantly greater in-hospital mortality and greater comorbidity. International Registry of Acute aortic Dissection data^20^ support this, with mortality increasing proportionally to the number of clinical malperfusions. Survival analysis confirmed the prognostic relevance of clinical malperfusion (M+). At 30 days, patients who were M+ exhibited significantly lower survival (log-rank *P* = .0149), highlighting the acute burden of systemic ischemia. Patients who were M+ continued to show lower long-term survival; however, this difference was no longer statistically significant at 10 years (log-rank *P* = .281). These findings suggest that the impact of malperfusion is primarily concentrated in the early postoperative period and may attenuate over time in long-term survivors. Territory-specific analysis showed consistent M+ mortality trends, although statistical significance was sometimes lacking, likely due to heterogeneity in definitions and smaller sample sizes.

Patients who were M3+ exhibited the greatest morbidity, including renal, pulmonary, visceral complications, and infections, as already described.^21^ These findings raise the question of whether M subgroups (except M1− and proximal aortic surgery) have sufficient clinical weight to justify distinction from M0. A simplified dichotomy (M0 vs M+) might clarify prognostic interpretation.

**Figure 2:**
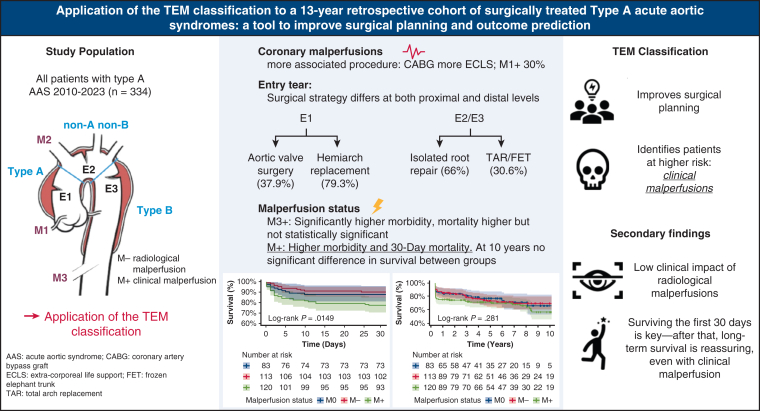
TEM classification: improving surgical planning and outcome prediction. *AAS*, Acute aortic syndrome; *CABG*, coronary artery bypass grafting; *ECLS*, extracorporeal life support.

Moreover, the current TEM malperfusion axis lacks some discriminative power. It does not differentiate between a pulse deficit and a large stroke, or between mild renal impairment and mesenteric ischemia, nor does it account for multiple insults within one territory. The TEM classification provides clinicians with an overview of the presence of malperfusions, their territories, and their impact, helping to stratify patients with worst expected outcomes, especially in patients who are M+, regardless of the topography (Figure 2).

### Limitations

This is a retrospective, single-center study spanning more than a decade, during which surgical practices evolved, such as systematization of open distal circulatory arrest repair, whereas limited ascending aortic replacement was mostly performed during the first years of the cohort. Surgical decisions were operator-dependent, and 21 surgeons were involved, which may introduce variability. As a high-volume center, our results may not generalize to all institutions. The relatively small number of patients in certain subgroups may have limited the statistical power of some analyses.

Selection bias is possible because only patients who underwent operation were included. Those who died before surgery or were deemed inoperable may have had more severe malperfusion, potentially underestimating its impact, explaining some of the conflicted results we have compared with the literature. For example, in other studies, coronary and neuro-malperfusions were found to be associated with a greater mortality.^22^ NonA-nonB forms were rare (1.5%), likely reflecting their frequent conservative treatment as type B dissections.

## Conclusions

The TEM classification, building on the Stanford system, is simple to apply and assists in surgical planning by identifying the proximal entry tear and high-risk patients, especially those with clinical malperfusion. Our findings support its adoption in clinical practice, as endorsed by recent European guidelines. Its main limitation lies in the lack of granularity regarding malperfusion symptoms. Combining it with a perfusion-focused system like the Penn classification may enhance risk stratification and guide decisions more precisely.

### Declaration of Generative AI and AI-Assisted Technologies in the Writing Process

During the preparation of this work the authors used ChatGPT (OpenAI) to assist with academic phrasing, syntax refinement, spelling correction, and formatting suggestions for publication according to the journal guidelines. After using this tool, the authors reviewed and edited the content as needed and take full responsibility for the content of the publication.

## Conflict of Interest Statement

The authors reported no conflicts of interest.

The *Journal* policy requires editors and reviewers to disclose conflicts of interest and to decline handling or reviewing manuscripts for which they may have a conflict of interest. The editors and reviewers of this article have no conflicts of interest.

## Figures and Tables

**Table E1:** Preoperative data

Characteristic	N = 334
Shock at arrival	70 (21%)
Tamponade	62 (19%)
Sinus rhythm	308 (94%)
Left ventricular ejection fraction	61.0 ± 7.3
Ascending aortic aneurysm	255 (83%)
Valsalva sinuses, mm	41.0 ± 7.9
Ascending aorta, mm	51.5 ± 8.7
Aortic regurgitation	180 (53.9%)

Values are n (%) or mean ± standard deviation.

**Table E2:** Perioperative data

Characteristic	N = 334
Diagnosis	
Dissection	293 (88)
IMH	32 (9.6)
Aortic rupture/ fissuration	7 (2.1)
Pseudoaneurysm	1 (0.3)
Aortic reparation	
Hemiarch	251 (76)
Total arche replacement	16 (4.8)
FET	19 (5.7)
Limited ascending aortic replacement	43 (13)
Wrapping	2 (0.6)
Associated endovascular procedure	
Proximal reparation	3 (0.9)
Isolated root reparation	204 (62)
Aortic valve replacement	32 (9.7)
Bentall	63 (19)
Tirone David	16 (4.8)
Florida sleeve	2 (0.6)
No reparation	13 (3.9)
Associated procedure	43 (13)
CABG or coronary reimplantation	33 (9.9)
Aortic valve reparation	5 (1.5)
Mitral valve replacement	1 (0.3)
Tricuspid annuloplasty	2 (0.6)
ASD closing	1 (0.3)
Lower extremity thrombectomy	1 (0.3)
Arterial canulation	
Femoral	170 (52)
Axillary	109 (33)
Central	46 (14)
Innominate artery	2 (0.6)
Right common carotid	1 (0.3)
Multiple	2 (0.6)
Venous canulation	
Femoral	42 (13)
Central	281 (85)
Bicaval	5 (1.5)
Multiple	2 (0.6)
Crossclamp time, min	123.5 ± 44.6
CPB time, min	182.0 ± 60.1
Circulatory arrest	
Circulatory arrest performed	297 (89)
Circulatory arrest time	37.1 ± 19.6
Arrest temperature	24.1 ± 4.3
Cerebral protection	
Deep hypothermia only	93 (31)
Retrograde	2 (0.7)
Antegrade unilateral	64 (22)
Antegrade bilateral	134 (45)
Retrograde + antegrade	3 (1.0)
Per-procedural complications	
ECLS	22 (6.6)
Death	5 (1.5)
Packing	17 (5.1)

Values are n (%) or mean ± standard deviation. *IMH*, Intramural hematoma; *FET*, frozen elephant trunk; *CABG*, coronary artery bypass grafting; *ASD*, atrial septal defect; *CPB*, cardiopulmonary bypass; *ECLS*, extracorporeal life support.

**Table E3:** Postoperative data

Characteristic	N = 329
Transfusion	
RBC units	258 (78)
Plasma	282 (85)
Plaquettes	277 (84)
Surgical revisions	
Total number of revisions	226
Number of patients concerned	155 (47.1)
Hemostasis∗	68 (30)
Cardiac tamponade∗	23 (10)
Pericardial drainage∗	50 (22)
Mechanical circulatory support∗	11 (5)
Major cardiac surgery (CABG or valve-related)∗	13 (5.7)
Mediastinitis∗	17 (7.5)
Scarpa revision∗	13 (5.7)
ECLS removal∗	8 (3.5)
Depacking∗	13 (5.7)
Mesenteric ischemia∗	6 (2.6)
Other∗	16 (7.1)
Complications	
Neurologic	
Stroke with persistent deficit	34 (11)
Stroke without persistent deficit	12 (3.7)
Spinal cord injury	8 (2.5)
Coma	7 (2.2)
Seizures	4 (1.2)
Anoxic brain injury	2 (0.6)
Peripheral lesions	4 (1.2)
Cardiogenic shock	30 (9.3)
Myocardial infarction	22 (6.8)
Prolonged ventilation	54 (16.2)
Pulmonary complications	71 (22)
Visceral complications	17 (5.3)
Renal failure	137 (42)
Dialysis	25 (7.7)
Infection	99 (31)
Arrhythmia	107 (33)
Heart block	14 (4.3)
Mortality	
30-d	53 (15.8)
In-hospital	55 (16.5)

Values are n (%). *RBC*, Red blood cells; *CABG*, coronary artery bypass grafting; *ECLS*, extracorporeal life support.

∗ Proportion of the total number of surgical revisions.

**Table E4:** Follow-up

Characteristic	N = 274
Patients reoperated	41 (15)
New intervention on the aorta	20 (7.3)
Other reintervention	
Valvular or coronary	14 (5.1)
Infectious endocarditis	3 (1.1)
Peripheral or abdominal aorta procedures	8 (2.9)
Postoperative	5 (1.8)

Values are n (%).

**Table E5:** Comparison of iterative aortic surgery based on the initial procedure

Group	Total	Reintervention
Aortic arch replacement	32	2 (6.2)
Hemiarch	225	16 (7.1)
*P* value		1

Values are n (%), χ^2^ test.

**Table E6:** Predicted versus observed mortality based on the GERAADA score

Group	Number of patients, n (%)	GERAADA score, %	Observed mortality, %	*P* value
<15% (low risk)	120 (38.6)	11.1	8.5	.4803
15%-30% (intermediate risk)	153 (45.8)	20.4	15.7	.1606
>30% (high risk)	51 (15.3)	41.9	35.3	.3954

Binomial test. *GERAADA*, German Registry of Acute Aortic Dissection type A.

**Table E7:** Outcomes based on the malperfusion status for each system

Outcomes	M1-0 N = 265	M1− N = 25	M1+ N = 26	*P* value∗
Time of hospitalization, d	18.3 ± 15.9	21.4 ± 17.4	23.8 ± 21.8	.6
Time in the ICU, d	8.9 ± 13.9	8.2 ± 7.4	12.8 ± 15.9	>.9
Stroke	37 (15)	3 (12)	2 (8.0)	.8
Renal failure	114 (45)	8 (32)	13 (52)	.3
Visceral complications	12 (4.7)	2 (8.0)	2 (8.0)	.4
Pulmonary complications	55 (22)	5 (20)	7 (28)	.7
Infection	75 (29)	7 (28)	12 (48)	.2
Death	40 (15)	4 (16)	6 (23)	.5

Outcomes	M2-0 N = 137	M2− N = 112	M2+ N = 67	*P* value∗
Time of hospitalization, d	18.0 ± 19.5	20.4 ± 14.3	18.6 ± 13.6	.065
Time in the ICU, d	8.3 ± 17.1	9.9 ± 10.6	9.7 ± 9.8	**.031**
Stroke	13 (10)	17 (16)	12 (18)	.2
Renal failure	56 (43)	52 (48)	27 (41)	.6
Visceral complications	4 (3.1)	9 (8.2)	3 (4.5)	.2
Pulmonary complications	23 (18)	23 (21)	21 (32)	.083
Infection	32 (25)	39 (36)	23 (35)	.12
Death	21 (15)	16 (14)	13 (19)	.6

Outcomes	M3-0 N = 134	M3− N = 131	M3+ N = 51	*P* value∗
Time of hospitalization, d	17.6 ± 19.8	19.4 ± 12.7	21.8 ± 16.1	**.005**
Time in the ICU, d	7.8 ± 17.3	9.6 ± 9.5	11.6 ± 11.9	**.004**
Stroke	17 (13)	19 (15)	6 (13)	>.9
Renal failure	46 (35)	57 (45)	32 (67)	**<.001**
Visceral complications	1 (0.8)	7 (5.5)	8 (17)	**<.001**
Pulmonary complications	23 (18)	28 (22)	16 (33)	.090
Infection	28 (22)	43 (34)	23 (48)	**.002**
Death	19 (14)	18 (14)	13 (25)	.13

Values are mean ± standard deviation or n (%). Bolded *P* values indicate statistical significance (*P* < .05). *ICU*, Intensive care unit.

∗ Kruskal-Wallis rank sum test; Fisher exact test.

## References

[1] Malaisrie S.C., Szeto W.Y., Halas M. (2021). 2021 the American Association for Thoracic Surgery expert consensus document: surgical treatment of acute type A aortic dissection. J Thorac Cardiovasc Surg.

[2] Daily P.O., Trueblood H.W., Stinson E.B., Wuerflein R.D., Shumway N.E. (1970). Management of acute aortic dissections. Ann Thorac Surg.

[3] Berretta P., Trimarchi S., Patel H.J., Gleason T.G., Eagle K.A., Di Eusanio M. (2018). Malperfusion syndromes in type A aortic dissection: what we have learned from IRAD. J Vis Surg.

[4] Sievers H.H., Rylski B., Czerny M. (2020). Aortic dissection reconsidered: type, entry site, malperfusion classification adding clarity and enabling outcome prediction. Interact Cardiovasc Thorac Surg.

[5] Czerny M., Grabenwöger M., Berger T. (2024). EACTS/STS guidelines for diagnosing and treating acute and chronic syndromes of the aortic organ. Eur J Cardiothorac Surg.

[6] DeBakey M.E., Henly W.S., Cooley D.A., Morris G.C., Crawford E.S., Beall A.C. (1965). Surgical management of dissecting aneurysms of the aorta. J Thorac Cardiovasc Surg.

[7] Dake M.D., Thompson M., van Sambeek M., Vermassen F., Morales J.P., DEFINE Investigators (2013). DISSECT: a new mnemonic-based approach to the categorization of aortic dissection. Eur J Vasc Endovasc Surg.

[8] Lombardi J.V., Hughes G.C., Appoo J.J. (2020). Society for Vascular Surgery (SVS) and Society of Thoracic Surgeons (STS) reporting standards for type B aortic dissections. J Vasc Surg.

[9] Augoustides J.G., Geirsson A., Szeto W.Y. (2009). Observational study of mortality risk stratification by ischemic presentation in patients with acute type A aortic dissection: the Penn classification. Nat Rev Cardiol.

[10] Patrick W.L., Yarlagadda S., Bavaria J.E. (2023). The Penn classification system for malperfusion in acute type A dissection: a 25-year experience. Ann Thorac Surg.

[11] Czerny M., Siepe M., Beyersdorf F. (2020). Prediction of mortality rate in acute type A dissection: the German Registry for Acute Type A Aortic Dissection score. Eur J Cardiothorac Surg.

[12] Howard C., Ponnapalli A., Shaikh S., Idhrees M., Bashir M. (2021). Non-A non-B aortic dissection: a literature review. J Card Surg.

[13] Christodoulou K.C., Karangelis D., Efenti G.M. (2023). Current knowledge and contemporary management of non-A non-B aortic dissections. World J Cardiol.

[14] Rylski B., Beyersdorf F., Kari F.A., Schlosser J., Blanke P., Siepe M. (2014). Acute type A aortic dissection extending beyond ascending aorta: limited or extensive distal repair. J Thorac Cardiovasc Surg.

[15] White A., Bozso S.J., Ouzounian M., Chu M.W.A., Moon M.C., Canadian Thoracic Aortic Collaborative (2021). Acute type A aortic dissection and the consequences of a patent false lumen. J Thorac Cardiovasc Surg Tech.

[16] Ma L., Chai T., Yang X. (2022). Outcomes of hemi- vs. total arch replacement in acute type A aortic dissection: a systematic review and meta-analysis. Front Cardiovasc Med.

[17] Jassar A.S., Sundt T.M. (2019). How should we manage type A aortic dissection?. Gen Thorac Cardiovasc Surg.

[18] Kamman A.V., Yang B., Kim K.M., Williams D.M., Michael Deeb G., Patel H.J. (2017). Visceral malperfusion in aortic dissection: the Michigan experience. Semin Thorac Cardiovasc Surg.

[19] Yang B., Rosati C.M., Norton E.L. (2018). Endovascular fenestration/stenting first followed by delayed open aortic repair for acute type A aortic dissection with malperfusion syndrome. Circulation.

[20] Wolfe S.B., Sundt T.M., Isselbacher E.M. (2024). Survival after operative repair of acute type A aortic dissection varies according to the presence and type of preoperative malperfusion. J Thorac Cardiovasc Surg.

[21] Czerny M., Schoenhoff F., Etz C. (2015). The impact of pre-operative malperfusion on outcome in acute type A aortic dissection: results from the GERAADA registry. J Am Coll Cardiol.

[22] Brown J.A., Aranda-Michel E., Navid F., Serna-Gallegos D., Thoma F., Sultan I. (2024). Outcomes of emergency surgery for acute type A aortic dissection complicated by malperfusion syndrome. J Thorac Cardiovasc Surg.

